# The adaptive physical activity programme in stroke (TAPAS): protocol for a process evaluation in a sequential multiple assignment randomised trial

**DOI:** 10.1136/bmjopen-2024-087016

**Published:** 2025-09-14

**Authors:** Padraic Rocliffe, Aoife Whiston, Amy O’ Mahony, Siobhán M O’Reilly, Margaret O’Connor, Nora Cunningham, Liam Glynn, Jane C Walsh, Cathal Walsh, Edel Hennessy, Eva Murphy, Andrew Hunter, Mike Butler, Lorna Paul, Claire F Fitzsimons, Ita Richardson, James G Bradley, Jon Salsberg, Sara Hayes

**Affiliations:** 1 School of Allied Health, Ageing Research Centre, Health Research Institute, University of Limerick, Limerick, Ireland; 2 Department of Ageing and Therapeutics, University Hospital Limerick, Limerick, Ireland; 3 School of Medicine, University of Limerick, Limerick, Ireland; 4 School of Psychology, University of Galway, Galway, Ireland; 5 School of Medicine, Trinity College Dublin, Dublin, Ireland; 6 Early Support Discharge, University Hospital Limerick, Limerick, Ireland; 7 School of Nursing and Midwifery, University of Galway, Galway, Ireland; 8 University of Limerick, Limerick, Ireland; 9 Glasgow Caledonian University School of Health and Life Sciences, Glasgow, UK; 10 Physical Activity for Health Research Centre, Institute Sport, Physical Education and Health Sciences, The University of Edinburgh, St Leonards Land, Holyrood Road, Edinburgh, EH8 8Aq, UK; 11 Department of Computer Science and Information Systems, University of Limerick, Limerick, Ireland; 12 Lero, The Science Foundation Ireland Research Centre for Software, University of Limerick, Limerick, Ireland; 13 School of Engineering, University of Limerick, Limerick, Ireland

**Keywords:** Health, Protocols & guidelines, STROKE MEDICINE, Cardiovascular Disease

## Abstract

**Abstract:**

**Introduction:**

Participation in physical activity (PA) is a cornerstone of the secondary prevention of stroke. Given the heterogeneous nature of stroke, PA interventions that are adaptive to individual performance capability and associated co-morbidity levels are recommended. Mobile health (mHealth) has been identified as a potential approach to supporting PA post-stroke. To this end, we used a Sequential Multiple Assignment Randomised Trial design to develop an adaptive, mHealth intervention to improve PA post-stroke – The Adaptive Physical Activity programme in Stroke (TAPAS) (Clinicaltrials.Gov NCT05606770). As the first trial in stroke recovery literature to use this design, there is an opportunity to conduct a process evaluation for this type of adaptive intervention. The aim of this process evaluation is to examine the implementation process, mechanism of change and contextual influences of TAPAS among ambulatory people with stroke in the community.

**Methods and analysis:**

Guided by the Medical Research Council Framework for process evaluations, qualitative and quantitative methods will be used to examine the (1) implementation process and the content of TAPAS (fidelity adaptation, dose and reach); (2) mechanisms of change (participants’ response to the intervention; mediators; unexpected pathways and consequences) and (3) influence of the context of the intervention. Quantitative data will be presented descriptively, for example, adherence to exercise sessions. Qualitative data will be collected among TAPAS participants and the interventionist using semi-structured one-to-one or focus group interviews. Transcribed interviews will be analysed using reflexive thematic analysis. Key themes and sub-themes will be developed.

**Ethics and dissemination:**

Ethical approval has been granted by the Health Service Executive Mid-Western Ethics Committee (REC Ref: 026/2022) (25/03/2024). The findings will be submitted for publication and presented at relevant national and international academic conferences.

**STRENGTHS AND LIMITATIONS OF THIS STUDY**

- This process evaluation will provide valuable insights into how adaptive physical activity (PA) programmes can be implemented for people with stroke.
- This study is the first of its kind to evaluate a sequential multiple assignment randomised trial design to develop and evaluate the optimum adaptive PA intervention for people with stroke.
- As The Adaptive Physical Activity programme in Stroke (TAPAS) intervention was delivered in the most part by one interventionalist, potential future implementation of the intervention across settings and interventionalists is warranted.
- Specific process evaluation protocols do not yet exist for SMART designs and future research should develop design specific evaluation criteria.

## Introduction

### Background

 Stroke accounts for over 11% of deaths worldwide and was found to be the third leading cause of mortality and disability globally. ^1^Meta-analytical evidence highlights that the 1-year risk of recurrent stroke is 11.1%, and this increases to 26.4% after 5 years.^2^ The targeting of certain modifiable risk factors, such as diabetes, obesity and hypertension, and lifestyle factors, such as physical activity (PA), has been shown to reduce the risk of recurrent stroke. O’Donnell *et al*^3^ undertook an international case control study (n=<26 000) and identified 10 key modifiable risk factors for stroke, with PA being the second largest predictor. There were variances in non-modifiable risk factors across countries, and therefore regional-specific and global programmes are recommended for stroke prevention.^4 5^ Despite the advancements made in stroke interventions for the acute stage, there is a significant lack of secondary prevention strategies. The American Heart Association guidelines for the prevention of stroke released in 2021 recommended that people with stroke should target population-based PA recommendations, or if unable to achieve this, their PA goals should be personalised to their stage of recovery, exercise tolerance preferences and social support.^6^ However, due to barriers such as poor health, physical impairments and environmental factors, up to 70% people with stroke have lower fitness levels than age-matched counterparts due to a lack of post-stroke PA.^7^

A sequence of personalised treatments is required for the effective clinical management of stroke, resulting in several decisions through a person’s rehabilitation journey.^8^ The empirical evidence to support optimal intervention sequence is lacking; however, trial designs wherein adaptive interventions are evaluated empirically may be beneficial. Sequential multiple Assignment Randomised Trials (SMARTs) allow for intervention sequences to be effectively assessed through the identification of responders and non-responders and intervention adaption according to one’s response. SMART designs were developed to build and provide robust evidence for adaptive interventions. SMARTs are factorial designs in a sequential setting^6^,^7^ and can be described as multistage RCT designs. Each stage corresponds to a decision point during the course of care. In this SMART design, participants are initially randomised once, and depending on response to the intervention, participants may be randomised again throughout the duration of the trial. Randomisation occurs at the beginning of the decision stages for all or some subset of participants. By enabling repeated randomisation of participants to treatments, the use of SMART designs allows for causal inference across decision points in an adaptive intervention.^9^. This may help to reduce the use of less effective interventions and identify larger benefits of PA treatments. SMARTs can be described as multistage randomised controlled trials that are factorial designs in a sequential setting.^8 9^

The Adaptive Physical Activity study for Stroke (TAPAS) used a SMART design to develop and evaluate an optimum adaptive PA intervention for people with stroke.^10^ This adaptive intervention included two intervention components: 1. Structured Exercise and 2. Lifestyle PA (ie, a set of videos on healthy lifestyle/PA habits) to increase PA, in addition to combinations of these treatment types. The objective of TAPAS, therefore, was to construct an adaptive PA intervention that would subsequently be evaluated against treatment-as-usual using a standard two-arm trial design. This allows the exploration of the optimum sequence of embedded treatments to improve PA in community-based people with stroke who are independently mobile.

TAPAS is considered a complex intervention. A complex intervention is one with multiple interacting components.^11^ The Medical Research Council (MRC) has provided guidance on the inclusion of process evaluations of complex interventions to gain a deeper understanding of the mechanisms of change.^11^,^13^ This guidance can help to explain discrepancies between expected and observed outcomes, highlight the complexities of an intervention and the impact of contextual factors on outcomes and thus to better inform implementation.^11 12^ The three key functions of process evaluations include: (1) examining the implementation process and its content (fidelity adaptation, dose and reach); (2) understanding the mechanisms of impact (participants’ response to the intervention, mediators and unexpected pathways and consequences) and (3) investigating the influence of the context of the intervention. In this process evaluation, we will use the MRC framework to evaluate the process, the delivery and impact of the implementation of the TAPAS intervention to improve PA in people with stroke in the community.

### Objectives

The aim of this process evaluation is to understand the functioning and effects of the TAPAS intervention that has been completed by examining how the intervention was delivered and received in practice. In line with the MRC guidelines for process evaluations of complex interventions,^11 12^ the process evaluation has the following objectives to achieve this aim: (1) to analyse the TAPAS trial implementation by examining delivery, fidelity, dose, reach and adaptions; (2) to explore mechanisms of impact, including barriers and facilitators to the impact of the intervention and (3) to identify contextual factors affecting impact, delivery and acceptability.

## Methods and Analysis

### Design

The process evaluation will employ a mixed-methods approach to address study objectives. The trial is registered on ClinicalTrials. Gov NCT05606770). The reporting of this protocol aligns with the Standard Protocol Items for Clinical Trials (SPIRIT) guidelines.^14^ A full reporting SPIRIT checklist is presented in the online supplemental material 1. In addition, the protocol is underpinned by components of the Criteria for Reporting the Development and Evaluation of Complex Interventions (CReDECI 2) in healthcare-revised guideline to account for the nature of the studies’ methodological design (ie, process evaluation, rather than a trial).^15^

### Participants

The evaluation will involve the interventionist who delivered the mHealth community-based TAPAS intervention and people with stroke who participated in TAPAS. Given the mHealth nature of the trial, delivered by one interventionist, and the fact that this was a feasibility trial, it is anticipated that around 20–25 participants will participate in focus groups/interviews following completion of the TAPAS trial. However, recruitment will continue until data saturation has been reached, as indicated by redundancy in the data and no new themes being generated.^16^ Potential participants will be identified by members of the research team directly involved in the intervention delivery. Participants will be eligible for inclusion if they are a person with stroke who participated in the TAPAS trial or if they delivered the TAPAS intervention. Prospective participants will be provided with an information sheet outlining the evaluation aim and procedure; written informed consent will be sought by the TAPAS postdoctoral researcher prior to participation. The interviews will take place when eligible participants have completed the intervention and follow-up assessments. The interviews will take place in the form of focus groups or individual interviews, according to participant preference. Participants in a focus group will not be randomised or grouped for intervention allocation.

### TAPAS intervention

**Figure 1:**
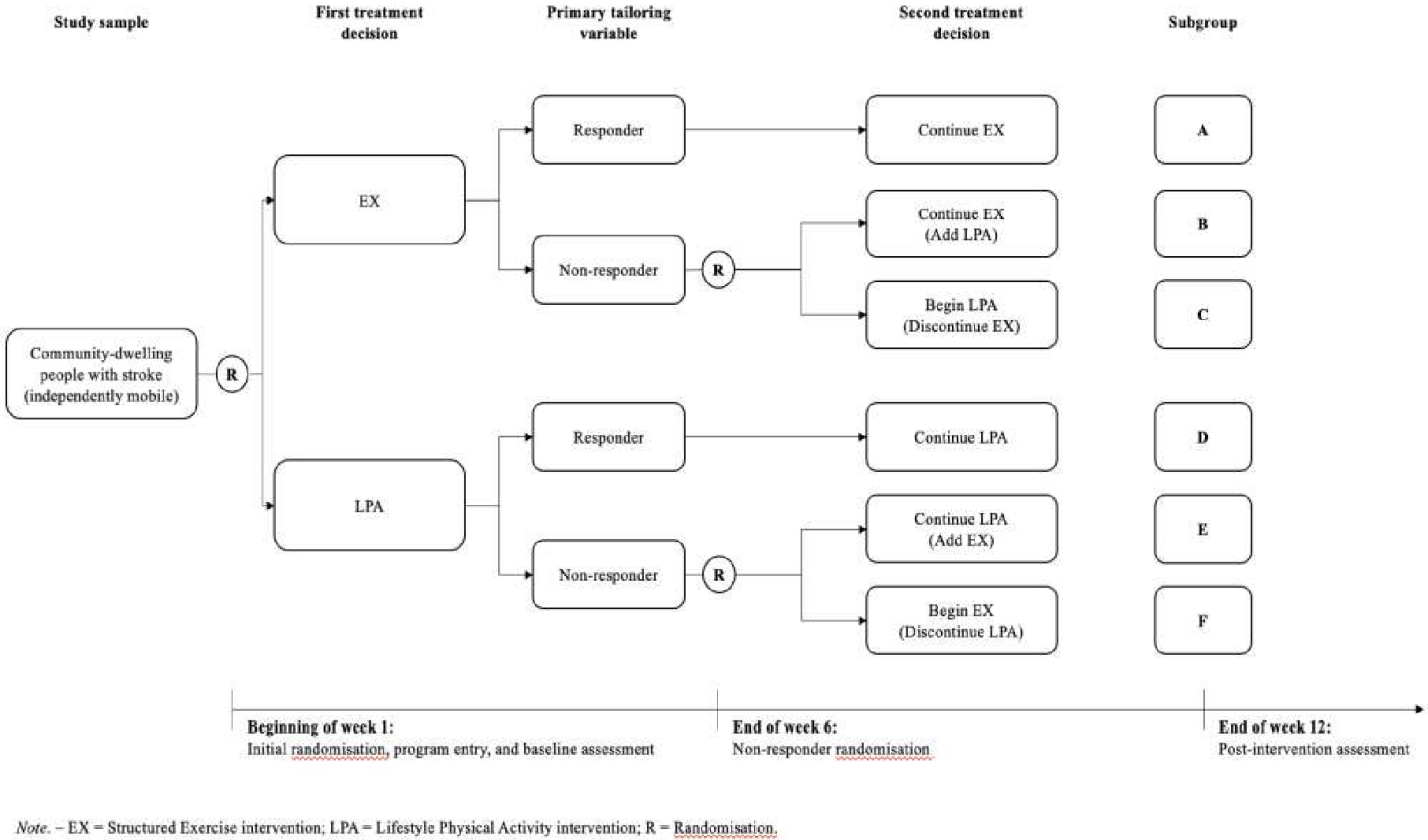
Sequential Multiple Assignment Randomised Trial design.

Figure 1 outlines the SMART design and the participant flow through the TAPAS intervention. There are two components that target increased PA in the TAPAS intervention: Structured Exercise and Lifestyle PA or a combination of both of these components. Common to both components of the intervention was the provision of a Fitbit Inspire 2 to all participants. This wrist-worn activity monitor allows for the measurement of steps, distance, calories and time spent engaged in activity. Real-time PA data were provided through a triaxial accelerometer, an altimeter, Global Positioning System and an optical heart-rate tracker. For the purpose of this trial, the Fitbit Inspire 2 was used to measure daily step count among participants and inform individualised step activity goals only. Goals were set based on individuals' own baseline walking and were advanced based on the achievement of previous goals. All participants were assigned a new step count goal weekly by adding 5% to the previous week’s 7-day average.

### Structured exercise intervention

In addition to the individualised step count intervention, participants assigned to the structured exercise component were provided with two times weekly strengthening exercise sessions, delivered through the digital platform. The individual exercise classes followed a circuit class style, with sessions gradually progressing in intensity throughout the programme. The structured exercise component was informed by international clinical guidelines^6 17^ and was prescribed by the TAPAS postdoctoral researcher. The full exercise intervention is published in the TAPAS protocol.^10^

### Lifestyle PA intervention

The Lifestyle PA component was developed using the behaviour change wheel (BCW) guide to designing interventions and is underpinned by the COM-B model of behaviour change.^18^ This posits that people need capability (C), opportunity (O) and motivation (M) to perform a behaviour (B). The aim of the Lifestyle PA component is to increase the capability, opportunity and motivation of participants to reach their daily step count goals. To achieve this, the three stages of the BCW intervention design process were followed. The first stage involved a review of the literature^19^ to identify the change objectives of the intervention. Stage two selected the intervention functions that would facilitate the changes to behaviour required to meet those objectives. The final stage defined the content of the intervention using behaviour change techniques (BCTs) and selected their mode of delivery. The full description of the Lifestyle PA intervention component is described in the TAPAS protocol.^10^

### Outcomes and measures

Using the MRC process evaluation framework, the study will focus on the measures and research questions outlined in table 1. Drawing from a similar approach to that of Cassarino et al (2019) (20), the process of implementation will be described in terms of activities and processes put in place for the development and delivery of the implementation, the fidelity of the intervention (adherence to protocol and evidence, as well as adaptations), its dose and reach. Mechanisms internal to the intervention will be investigated in relation to the participants’ interaction with the intervention, potential mediators and unexpected pathways. Finally, using a system approach, potential facilitators and barriers to implementation outside of the intervention will be explored at the level of individuals.

**Table 1:** Measures, research questions and data collection

Dimension	Measure	Research question	Data source	Analysis type
Implementation	Process Outcomes	How were the interventions delivered? What was the processing time for enrolling participants?	Activity logs and TAPAS smartphone application data analytics Recruitment logs	Quantitative descriptive Quantitative descriptive
	Fidelity of receipt	Was the intervention delivered as expected? Were participants adhering to all aspects of the intervention?	Activity logs and TAPAS smartphone application data analytics Activity logs	Quantitative descriptive Quantitative descriptive
	Dose	What was the attrition rate? What was the frequency, duration and type of exercise within the intervention?	Recruitment logs Activity logs and TAPAS smartphone application data analytics	Quantitative descriptive Quantitative descriptive
	Reach	Was the correct population targeted?	Recruitment logs	Quantitative descriptive
Mechanisms	Interaction with the intervention	What were the participants’ and interventionist experiences of being part of the intervention?	Focus groups/structured interview (with participants/interventionist)	Qualitative
	Barriers and facilitators	What factors/mechanisms affected or impacted participants’ involvement and adherence to the intervention?	Focus groups/structured interviews	Qualitative
Context	Impact and acceptability	What did participants gain from being in the trial? Do participants feel this intervention is wanted by people with stroke? What recommendations would participants and interventionists make for a future definitive trial? How could it be improved? Should the intervention be considered for the reduction of risk of secondary stroke in the future?	Focus groups/structured interviews Focus groups/structured interviews Focus groups/structured interviews Focus groups/structured interviews	Qualitative Qualitative Qualitative Qualitative

Based on the table outlined in Cassarino *et al*^21^ based on the MRC guidance^13^

TAPAS, The adaptive physical activity programme in stroke.

### Data collection and analysis

All electronic and hardcopy data will be stored safely by the research team and retained in accordance with the data management policies and procedures of the University of Limerick, Ireland. Access to the data will be limited to the research team members involved in the data analysis. Participants will be assigned a unique participant number when the focus groups are transcribed. A separate password-protected Excel file will hold participants' details and their unique participant number on a password-protected laptop. Audio files will be destroyed after being transcribed and the research team will only have access to anonymised transcripts. These transcripts and descriptive statistics will be stored on a password-protected laptop. Transcripts of focus groups will not be offered to participants, but summaries will be provided on request. Consent forms and data collection forms will be stored on-site in the School of Allied Health in SH’s locked office in a locked cabinet. All data will be pseudonymised and stored in accordance with GDPR.

As described in table 1 (Cassarino et al (2019) (20), a mix of quantitative and qualitative methods will be used to address the objectives of this process evaluation. The content and process of delivery will be evaluated quantitatively through the mHealth platform web analytics software. The implementation will also be investigated in terms of fidelity, dose and reach (e.g., overall number of videos watched). For the purpose of this process evaluation, we will integrate a quantification of overall programme adherence (e.g., overall adherence to Fitbit wearing), dose and reach with a qualitative exploration of mechanisms of impact within and beyond the intervention.

### Fidelity outcomes

According to the fidelity framework outlined by the US National Institute of Health Behaviour Change Consortium, there are five fidelity dimensions.^20^ These are design, training, delivery, receipt and enactment.^21^ The fidelity of intervention design relates to the acceptability of interventions and how these interventions are specified a priori.^20^ The intervention delivered in the TAPAS trial was based on qualitative findings and was then reviewed by the associated Patient and Public Involvement (PPI) panel. While designing the intervention, experts in the area of stroke provided feedback and suggestions on the content to ensure both an evidence-based approach and clinical face validity. The intervention delivery parameters (i.e., dose/duration/number of contacts) and content (i.e., component BCTs) of the TAPAS trial were specified in detail.^10^ The content, specifically step-count goals, was personalised to individual progress of participants throughout the duration of the 12-week intervention. Fidelity of training refers to the competency and training of interventionalists to deliver interventions.^20^ The TAPAS trial was mHealth-based with most intervention components available on the online platform, and training was provided to the interventionist on assigning programmes, goals and for the lifestyle PA group scripted phone calls and text communication. Website analytics will be used to assess fidelity of receipt of all programme components. They will be used to determine if the participants completed their tasks, watched the videos assigned to them (for the lifestyle PA groups) and if they have taken part in the phone calls. Step count will be measured using the Fitbit Inspire 2 to assess fidelity of enactment.

Potential modifications will be quantified and described by the interventionist using intervention logs for each participant. The qualitative elements of the implementation will be explored via semi-structured interviews and focus groups. An interview schedule is presented in the online supplemental material 2 with questions tailored to the interventionist and people with stroke. Prospective participants who do not wish or are not able to take part in the focus groups will be invited to participate in 1:1 semi-structured interviews.

The interviews and focus groups will be recorded, anonymised and transcribed verbatim by the TAPAs postdoctoral researcher. Reflexive thematic analysis will be conducted in accordance with the six steps outlined by Braun and Clarke^22^ (i) data familiarisation, involving repeated active engagement with notes and transcripts; (ii) generation of initial codes; (iii) conceptualisation of themes, involving the identification and interpretative analysis of the collated codes (iv) reviewing and refinement of themes; (v) defining and naming of themes; (vi) producing the final report. A qualitative descriptive approach will be used^23^ and findings will be reported in accordance with the COnsolidated criteria for REporting Qualitative research checklist to ensure rigour.^24^ NVivo^25^ will be used to check the accuracy of the themes. Executive summary statements will be developed as the foundation for drafting the findings that will be described and explain the active ingredients of the intervention from the perspective of the participants. This will also serve to identify key issues, which may need to be addressed in advance of a future full-scale trial. Access to the data will be limited to the research team members involved in data analysis (PR, SH and AW).

### Patient and public involvement statement

The TAPAS project PPI group, including people post-stroke and multidisciplinary healthcare professionals, is already in place and actively contributing to the programme of research. The PPI panel members are actively involved as co-researchers in the conceptualisation of this process evaluation and will be involved in the delivery, analysis, interpretation and dissemination of this research.

## Discussion

Process evaluations have emerged as a vital component in investigations of the efficacy of health interventions, progressively gaining significance within investigative frameworks.^13^ The current paper provides a detailed protocol for a process evaluation underpinned by a SMART of an adaptive PA intervention in people with stroke. Despite the progress achieved in acute-stage stroke interventions, a paucity of evidence exists concerning secondary prevention strategies. The current process evaluation protocol represents the first in trial stroke recovery literature using this design, thereby offering an opportunity to examine the implementation process, mechanism of change and contextual influences among ambulatory individuals with stroke in the community. Using the MRC framework for process evaluations offers a structured approach to examining the development, feasibility, evaluation and implementation of the TAPAS intervention, while also comprehensively capturing the nuances of the intervention for both people with stroke in the community in parallel with the interventionist involved in the conceptualisation and implementation of the project.^11^,^13^ Furthermore, what constitutes effective implementation concerning the practical components of a complex project with a heterogenous population of people with stroke will be established, proving to be a pivotal consideration for practitioners, researchers and the development of both future recommendations concerning process orientated research and public health policy development in relation to post-stroke management.

### Ethics and dissemination

Ethical approval has been granted by the Faculty of Education and Health Sciences Research Ethics Committee at the University of Limerick (2022_02_15_EHS (OA)) and the HSE Mid-Western Ethics Committee (REC Ref: 026/2022). The findings will be submitted for publication and presented at relevant national and international academic conferences. A simplified version of the findings will be presented to interested participants and to the PPI group associated with the study.

## Supplementary material

10.1136/bmjopen-2024-087016 online supplemental file 1

10.1136/bmjopen-2024-087016 online supplemental file 2

## Data Availability

No data are available.
