# Supplementary material for: The adaptive physical activity programme in stroke (TAPAS): protocol for a process evaluation in a sequential multiple assignment randomised trial
Source: BMJ Open. 2025 Sep 14;15(9):e087016. doi: 10.1136/bmjopen-2024-087016 (PMC12434737; doi:10.1136/bmjopen-2024-087016)
Supplement: online supplemental file 2 [file bmjopen-15-9-s002.docx]

## **Supplementary Material 2**

**Process evaluation protocol**

**Interview script- internationalist**

**Fidelity and quality of Implementation**

Tell me about the preparation phase prior to implementation of the Sequential Multiple Assignment Randomised Trial (SMART): What types of activities were carried to prepare for the trial?

Could you describe a typical day of the intervention?

Do you think that the implementation was carried as planned or were there adaptations made? If so, which?

How did you feel about the recruitment part of the SMART?

How did you feel about the tailoring variable and the re-randomisation part of the SMART?

How did you feel about the different modes of intervention delivery? For example, phone calls, daily texts, smartphone application, wearable sensor feedback.

How did you feel about the reach of the intervention- in terms of the participants who were participating in the intervention.

**Mechanisms**

How do you think the participants felt about being involved in the intervention?

What, in your opinion, worked well in the intervention and what might have worked better?

What is the impact of participation on you professionally?

Do you recall anything unexpected occurring during the intervention that might have influenced its implementation?

**Context**

What factors external to the intervention do you think have influenced its implementation and participant outcome?

**Recommendations for future practice**

What recommendations would you make for a future, definitive trial?

How do you think it can be improved?

Should the intervention be considered for the reduction of risk of secondary stroke in the future?

Is there anything else that you would like to add that you think is important about your experience and/or needs?

**Process evaluation protocol**

**Interview script- SMART participant**

**Fidelity and quality of Implementation**

Tell me about the preparation phase prior to starting the TAPAS programme. What types of activities were carried to prepare you for the trial?

How did you feel about the recruitment part of TAPAS? What more could have been done to inform you?

How did you feel about the "onboarding”/initial visit with the TAPAS researcher?

Could you describe a typical day of the TAPAS programme? What was involved on a daily and weekly basis?

Do you think that the TAPAS programme was carried out as planned or were there adaptations made? If so, which?

Can you describe how you accessed the TAPAS programme and took part in it?

If the nature of your TAPAS intervention switched during the programme, how did you feel about the change/ tailoring?

 How did you feel about the different modes of intervention delivery? For example, phone calls, daily texts, smartphone application, wearable sensor feedback.

How did you feel about the “reach” of the TAPAS intervention- do you think the people who need it would access it?

**Mechanisms**

How did you feel about being involved in the TAPAS intervention?

What, in your opinion, worked well in the TAPAS intervention and what might have worked better?

What is the impact of participation on you personally?

Do you recall anything unexpected occurring during the TAPAS intervention that might have influenced its implementation?

**Context**

What factors external to the TAPAS intervention do you think have influenced its implementation and your outcome? For example, your specific context/situation.

**Recommendations for future practice**

What recommendations would you make for a future, definitive trial?

How do you think it can be improved?

Should the intervention be considered for the reduction of risk of secondary stroke in the future?

Is there anything else that you would like to add that you think is important about your experience and/or needs?
