# Supplementary material for: The adaptive physical activity programme in stroke (TAPAS): protocol for a process evaluation in a sequential multiple assignment randomised trial
Source: BMJ Open. 2025 Sep 14;15(9):e087016. doi: 10.1136/bmjopen-2024-087016 (PMC12434737; doi:10.1136/bmjopen-2024-087016)
Supplement: Supplementary file 3 [file bmjopen-2024-087016.reviewer_comments.pdf]

PEER REVIEW HISTORY

BMJ Open publishes all reviews undertaken for accepted manuscripts. Reviewers are asked to complete a checklist review form and are provided with free text boxes to elaborate on their assessment. These free text comments are reproduced below.

ARTICLE DETAILS

Title (Provisional)

The Adaptive Physical Activity programme in Stroke [TAPAS]: protocol for a process evaluation in a Sequential Multiple Assignment Randomised Trial

Authors

Rocliffe, Padraic; Whiston, Aoife; O' Mahony, Amy; O'Reilly, Siobhan; O'Connor, Margaret; Cunningham, Nora; Glynn, Liam; Walsh, Jane C.; Walsh, Cathal; Hennessy , Edel; Murphy , Eva; Hunter , Andrew; Butler , Mike; Paul, Lorna; Fitzsimons, Claire F.; Richardson, Ita; Bradley, James G.; Salsberg, Jon; hayes, sara

---

VERSION 1 - REVIEW

**Reviewer**	**1**
**Name**	**Cabanas-Valdés, Rosa**
**Affiliation**	**Universitat Internacional de Catalunya Facultat de Medicina i Ciencies de la Salut, Physiotherapy**
**Date**	**30-May-2024**
**COI**	**I have no conflict of interest**

This a protocol for a process evaluation in a Sequential Multiple Assignment Randomised Trial, in my opinion it is well written.

**Reviewer**	**2**
**Name**	**Hsieh, Cheng**
**Affiliation**	**Sin Lau Christian Hospital**
**Date**	**15-Sep-2024**
**COI**	**Nil.**

Overall, this protocol has been well-written. I have only minor comments for revision.

First, compared to regular randomized controlled trials, the process of Sequential Multiple Assignment Randomized Trials (SMARTs) may be less known by the readers. I recommend the authors provide an example illustrated by a figure regarding how SMARTs work. How can

SMARTs achieve the goal of being able to "allow for intervention sequences to be effectively assessed through the identification of responders and non-responders and intervention adaption according to one's response"?

Second, the Background section seems too lengthy. I suggest to move some contents to the section of Methods and Analysis. That is, add a new sub-section of intervention between sub-sections of "Participants" and "Outcomes and measures".

Third, regarding the sub-sections of Participants, I suggest to list the details of inclusion and exclusion criteria for the present study. Besides, can the 20-25 participants you have planned provide a sufficient statistical power for the present study? How did you derive that number (ie, 20-25) ?

Fourth, in Page 12 & 14, what did the term "PPI" mean?

Fifth, in Page 15, the recruitment period was between March to April, 2024. Is a recruitment period of one month enough to recruit the anticipated number of participants?

**Reviewer**	**3**
**Name**	**Bertoni , Rita**
**Affiliation**	**Fondazione Don Carlo Gnocchi Onlus**
**Date**	**31-Jan-2025**
**COI**	**I do not have any competing interest.**

The present work describes an interesting protocol for the process evaluation of a sequential multiple assignment randomised trial aimed at improving physical activity in ambulatory persons with stroke. Combining quantitative and qualitative data originating from the study participants can bring unexpected improvements to intervention protocols, especially in rehabilitation, where people are the main actors. Unfortunately, this approach is still underused. The work is well written and presented.

However, I have only a few minor comments for the authors:

1. Tapas intervention: The first sentence about the flow of participants refers to Table 1, which I assume should be Figure 1, which is not included in the current version.

2. Methods and Analysis: Is it not clear (maybe is specified in the table/figure missing when the interviews will take place? Will the focus group be randomised or grouped for intervention allocation?

3. Fidelity outcomes: Please specify the acronym PPI as it is not yet known worldwide.

VERSION 1 - AUTHOR RESPONSE

**Reviewer 1 Comments**	**Authors Responses**
This a protocol for a process evaluation in a Sequential Multiple Assignment Randomised Trial, in my opinion it is well written.	Thank you for this feedback, it is greatly appreciated.
**Reviewer 2 Comments**	**Authors Responses**
I recommend the authors provide an example illustrated by a figure regarding how SMARTs work	Many thanks. Please see Figure 1 which is now included in the "TAPAS intervention" section of the manuscript- this outlines the SMART design used in the TAPAS trial.
How can SMARTs achieve the goal of being able to "allow for intervention sequences to be effectively assessed through the identification of responders and non-responders and intervention adaption according to one's response"?	Thank you- to clarify we have added the following to the 2nd paragraph of the background: "SMART designs were developed to build and provide robust evidence for adaptive interventions. SMARTs are factorial designs in a sequential setting[6,7] and can be described as multistage RCT designs. Each stage corresponds to a decision point during the course of care. In this SMART design, participants are initially randomised once, and depending on response to the intervention, participants may be randomised again throughout the duration of the trial. Randomization occurs at the beginning of the decision stages for all or some subset of participants. By enabling repeated randomization of participants to treatments, the use of SMART designs allows for causal inference across decision points in an adaptive intervention (Whiston et al 2025)."
The Background section seems too lengthy. I suggest to move some contents to the section of Methods and Analysis. That is, add a new sub-section of intervention between sub-sections of "Participants" and "Outcomes and measures".	Many thanks- we have moved the detail on the intervention to a new sub-section "TAPAS Intervention" in the methods section.
Regarding the sub-sections of Participants, I suggest to list the details of inclusion and exclusion criteria for the present study. Besides, can the 20-25 participants you have planned provide a sufficient statistical power for the present study? How did you derive that number (ie, 20-25) ?	Thank you, we have included the following to the "participants"section of the methods section "Participants will be eligible for inclusion if they: are a person with stroke who participated in the TAPAS trial or if they delivered the TAPAS intervention."<br>Relating to sample size we have added the following further detail, which will enable us to

	derive sufficient data "However, recruitment will continue until data saturation has been reached, as indicated by redundancy in the data and no new themes being generated (Saunders et al 2018)."
In Page 12 & 14, what did the term "PPI" mean?	Many thanks for this feedback. PPI stands for Patient and Public Involvement. This has now been written in full upon first usage on page 12 of the manuscript.
In Page 15, the recruitment period was between March to April, 2024. Is a recruitment period of one month enough to recruit the anticipated number of participants?	Many thanks for this feedback. You are correct, this has now been amended to illuminate a 2-month period for recruitment between March and May 2024.
**Reviewer 3 Comments**	**Author Responses**
The work is well written and presented.	Many thanks for this feedback, it is greatly appreciated.
Tapas intervention: The first sentence about the flow of participants refers to Table 1, which I assume should be Figure 1, which is not included in the current version.	Many thanks. Please see Figure 1 which is now included in the "TAPAS intervention" section of the manuscript- this outlines the SMART design used in the TAPAS trial.
Methods and Analysis: Is it not clear (maybe is specified in the table/figure missing when the interviews will take place? Will the focus group be randomised or grouped for intervention allocation?	Thank you- the following has been added to the "Participants" section:<br>"The interviews will take place when eligible participants have completed the intervention and follow-up assessments. At the time of submission of this protocol, participant recruitment was ongoing and was expected to be completed by the end of 2024. The interviews will take place in the form of focus groups or individual interviews, according to participant preference. Participants in a focus group will not be randomised or grouped for intervention allocation."
Fidelity outcomes: Please specify the acronym PPI as it is not yet known worldwide.	Many thanks for this feedback. PPI stands for Patient and Public Involvement. This has now been written in full upon first usage on page 12 of the manuscript.

VERSION 2 - REVIEW

**Reviewer**	**2**
**Name**	**Hsieh, Cheng**
**Affiliation**	**Sin Lau Christian Hospital**
**Date**	**07-Jun-2025**
**COI**	

I would judge this revision as fully responsive to prior comments and suitable for acceptance

**Reviewer**	**3**
**Name**	**Bertoni , Rita**
**Affiliation**	**Fondazione Don Carlo Gnocchi Onlus**
**Date**	**26-May-2025**
**COI**	

Thank you for providing the revised manuscript. All the comments have been addressed correctly. In my opinion, the protocol is clear in all its aspects.
